# Sarcopenia predicts an adverse prognosis in patients with combined hepatocellular carcinoma and cholangiocarcinoma after surgery

**DOI:** 10.1002/cam4.4448

**Published:** 2021-12-05

**Authors:** Gui‐min Hou, Chuang Jiang, Jin‐peng Du, Ke‐fei Yuan

**Affiliations:** ^1^ Department of Liver Surgery & Liver Transplantation State Key Laboratory of Biotherapy and Cancer Center West China Hospital Sichuan University and Collaborative Innovation Center of Biotherapy Chengdu China

**Keywords:** clinical‐pathological features, combined hepatocellular carcinoma and cholangiocarcinoma, prognostic model, sarcopenia

## Abstract

**Background:**

The prognostic value of sarcopenia in combined hepatocellular carcinoma and cholangiocarcinoma (cHCC‐CC) patients after surgery has not been evaluated, while the efficacy of the available tumor stage for cHCC‐CC remains controversial.

**Methods:**

All consecutive cHCC‐CC patients after surgery were retrieved. The patients were stratified by the sex‐specific medians of the psoas muscle index into groups with or without sarcopenia. Prognosis was analyzed using the Kaplan–Meier (K–M) method, and the K–M curves were adjusted by inverse probability weighting (IPW). A nomogram based on Cox regression analysis was established and further compared with primary liver cancer (PLC) stages by internal validation based on bootstrap resampling and k‐fold cross‐validation.

**Results:**

A total of 153 patients were stratified into sarcopenia and non‐sarcopenia groups. The sarcopenia group revealed statistically worse overall survival (OS) and disease‐free survival (DFS) using the K–M method and K–M curves adjusted by IPW. Multivariate Cox regression analyses suggested sarcopenia as an independent risk factor for OS (HR = 1.55; *p* = 0.040) and DFS (HR = 1.55; *p* = 0.019). Subgroup analysis based on baseline variables showed sarcopenia as a stable risk factor for the prognosis. Our nomogram outperformed PLC stages in prognostic prediction, as evidenced by the best c‐index, area under the curve, and positive improvement of the net reclassification index and integrated discrimination improvement. A fivefold cross‐validation revealed consistent results. Decision curve analysis revealed higher net benefits of the nomogram than PLC stages.

**Conclusions:**

Sarcopenia is an independent and stable risk factor for the prognosis of cHCC‐CC patients after surgery. Our nomogram might aid high‐risk patient identification and clinical decisions.

## Graphical abstract

Sarcopenia, defined by the PMI, which was objective and easy to measure, was an independent and stable adverse prognostic factor in cHCC‐CC patients after surgery. Nomogram based on sarcopenia and clinicopathological characteristics might aid high‐risk patient identification and clinical decisions.

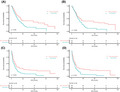

## 1 INTRODUCTION

Combined hepatocellular carcinoma and cholangiocarcinoma (cHCC‐CC) is a distinct subset of primary liver cancer (PLC) in addition to hepatocellular carcinoma (HCC) and intrahepatic cholangiocarcinoma (ICC), sharing both hepatocytic and biliary differentiation and displaying a more dismal prognosis than HCC and ICC.^1^, ^2^, ^3^ However, because of its low prevalence and insufficient focus, its clinical characteristics and prognostic factors remain unclear.^4^, ^5^, ^6^

Available tumor stages for PLC, such as the tumor–node–metastasis (TNM) stage^7^ for intrahepatic cholangiocarcinoma (ICC‐TNM), TNM stage for hepatocellular carcinoma (HCC‐TNM), and Barcelona Clinic Liver Cancer (BCLC) stage,^8^ have been established to assess the prognosis and guide the therapeutic approach, which always incorporates variables concerning the anatomic extent of disease and liver function. However, accumulating studies have revealed poor prognostic prediction of conventional PLC stages for cHCC‐CC patients,^9^, ^10^, ^11^ probably resulting from unappreciation and neglect of the cancer patients’ general state.

Emerging evidence has reported sarcopenia as an independent risk factor for a poor prognosis in various malignancies, including esophageal cancer,^12^ colorectal cancer,^13^ non‐small cell lung cancer,^14^ pancreatic cancer,^15^ and adverse outcomes for patients following hepatectomy or liver transplantation.^16^, ^17^, ^18^ However, the prognostic value of sarcopenia in cHCC‐CC patients after surgery has not been evaluated.

Therefore, this study aimed to evaluate whether sarcopenia is a poor prognostic factor and establish a comprehensive prognostic model to aid high‐risk group identification and clinical decisions for surgically treated cHCC‐CC patients.

## 2 MATERIALS AND METHODS

### 2.1 Patients

This study was approved by the ethics committee of West China Hospital. Between 2000 and 2018, consecutive patients with pathologically confirmed cHCC‐CC based on histological and immunohistochemical staining^19^ after hepatectomy for PLC at West China Hospital were identified. The exclusion criteria were as follows: (1) neoadjuvant therapy was received preoperatively, such as chemotherapy, targeted therapy, transarterial chemoembolization (TACE), and radiofrequency ablation (RFA); (2) CT scan images within 1 month before surgery were not available; (3) emergency surgery was performed; (4) patients had undergone liver transplantation; and (5) patients had died within 1 month after surgery.

### 2.2 Data collection

The clinical and pathological data were extracted from the medical records, surgical records, and pathological reports and included the following: age, sex, body mass index (BMI), preoperative serum levels of alpha‐fetoprotein (AFP), carcinoembryonic antigen (CEA), carbohydrate antigen 19‐9 (CA19‐9), HBV or HCV infection, nonalcoholic steatohepatitis (NASH), alcoholic hepatitis, cirrhosis, tumor number, tumor size (cm), Child–Pugh classification, anatomic resection, differentiation, type of resection, minimally invasive surgery (MIS), lymph node (LN) metastasis, vascular invasion (VI), margin status, capsule involvement, satellite nodule status, HCC‐TNM 8th stage, ICC‐TNM 8th stage, and BCLC stage. Major hepatectomy was defined as resection of at least three Couinaud segments^20^. The cut‐off values for AFP, CEA, and CA19‐9 were 8, 3.4 ng/ml, and 22 U/ml, respectively, which were the reference upper limit values of our institution. BMI was calculated as the weight (kg)/height (m^2^) and was categorized as underweight (BMI < 18.5), normal (18.5 < BMI < 24.0), overweight (24 < BMI < 28), and obese (BMI > 28).^21^ Overall survival (OS) was defined as the duration between surgery and date of death or last follow‐up. Disease‐free survival (DFS) was defined as the duration between surgery and the date of recurrence or last follow‐up.

### 2.3 Sarcopenia and image analysis

Considering the Asian Working Group guidelines for Sarcopenia (AWGS)^22^ and previous reports,^23^, ^24^ the psoas muscle index (PMI) was applied to identify sarcopenia in our study. PMI is defined as the psoas muscle area (PMA) at the third lumbar vertebrae (L3) level in axial imaging divided by the height squared.^24^ PMA was calculated as follows: PMA = a × b × π, where "*a*" and "*b*" are the radii of the major and minor axes, respectively.^23^ Anonymous abdominal CT for skeletal muscle within 1 month before surgery was analyzed by two trained independent investigators. We defined the sex‐specific median as the cut‐off value in our study to establish the cHCC‐CC‐specific assessment for sarcopenia.^25^

### 2.4 Follow‐up

Patients were regularly followed up every 3 months in the first 2 years and every 6 months thereafter. Routine blood tests, liver function, CEA/AFP/CA19‐9 level measurement, and imaging examination (liver ultrasonography, CT, or magnetic resonance imaging) were performed at each follow‐up visit. The last follow‐up date was March 1, 2021.

### 2.5 Statistical analysis

Continuous variables were described as medians with interquartile ranges (IQRs) or means with standard deviation (SD) and were analyzed using the *t*‐test or the Mann–Whitney *U* test, while categorical variables were presented as frequencies and percentages and were compared by the Pearson's chi‐squared test or the Fisher's exact test as appropriate. We constructed a propensity score for sarcopenia exposure using a logistic regression, applied inverse probability weighting (IPW) to adjust potential confounders between the non‐sarcopenia and sarcopenia groups.^26^ The Kaplan–Meier curves with the log‐rank tests and K–M curve adjusted by IPW were applied for survival analysis between strata. A univariate Cox proportional hazards model was used to screen potential predictors of prognosis; variables with *p* < 0.1 and clinical relevance were incorporated into multivariate analyses. The prognostic nomogram was established based on multivariate analyses by backward stepwise selection using the Akaike Information Criterion (AIC).^27^ The prognostic nomogram and PLC stages were compared using the concordance index (c‐index), area under the curve (AUC) values of time‐dependent receiver operating characteristics (td‐ROC),^28^ net reclassification index (NRI),^29^, ^30^ integrated discrimination improvement (IDI),^30^ and decision curve analysis (DCA).^31^ The integrated AUC was defined as the average AUC of the first 60 months after surgery.^32^ The comparison was further internal validated by K‐fold cross‐validation to address concerns of overfitting.^33^ The overall performance of models was evaluated by Brier score, in which 0 indicates a perfect model and 0.25 represents uninformative model.^34^ Bilateral tests were used for all statistical tests, and a *p* value <0.05 was considered statistically significant. Statistical analyses were performed using SPSS Statistics (version 23.0; IBM Corporation) and R software (version 4.1.0).

## 3 RESULTS

A total of 182 patients were initially identified; 29 patients were excluded, and the remaining 153 patients were included in our study (Figure S1).

### 3.1 Baseline characteristics of the entire cohort and stratification by the cut‐off value of the PMI

There was no statistical difference in baseline characteristics between observed patients with and without PMI information (Table S1), so further analyses excluded the latter. Table S2 shows the baseline characteristics of the enrolled patients. A predisposition to male sex and HBV infection was noticeable in our cohorts. The schematic diagram of PMA is delineated in Figure S2. The median PMI values with IQR for men and women were 5.42 (4.50–7.01) cm^2^ F/m^2^ and 4.05 (3.44–4.31) cm^2^/m^2^, respectively. To establish the cHCC‐CC‐specific assessment for sarcopenia, we used the sex‐specific medians as cut‐off values to stratify our cohort into the non‐sarcopenia group (*n* = 76) and sarcopenia group (*n* = 77). Variables were comparable between the two groups regarding the performance status, differentiation, satellite nodule status, resection type, minimally invasive surgery, tumor size, tumor number, capsule involvement, margin status, LN metastasis, HBV infection, tumor marker levels, and Child–Pugh classification. The distribution of the non‐sarcopenia and sarcopenia patients in PLC stages was also comparable. Sarcopenia was significantly more common in patients aged 55 years or older (*p* = 0.027), with cirrhosis (*p* = 0.028), with VI (*p* = 0.027), and with a lower BMI (*p* < 0.001) (Table 1).

**TABLE 1:** Comparison of the clinicopathological factors of patients with non‐sarcopenia and sarcopenia

Variables	Non‐sarcopenia (*n* = 76, %)	Sarcopenia (*n* = 77, %)	*p* value
PMI, cm^2^/m^2^	6.70 (5.88–7.92)	4.31 (3.57–4.81)	<0.001
Age (years)			0.027
≤55	49 (64.47%)	36 (46.75%)	
>55	27 (35.53%)	41 (53.25%)	
Gender			0.855
Male	64 (84.21%)	64 (83.12%)	
Female	12 (15.79%)	13 (16.88%)	
Performance status			0.369
0	60 (78.95%)	56 (72.73%)	
1–2	16 (21.05%)	21 (27.27%)	
Differentiation			0.819
Well/moderately	51 (67.11%)	53 (68.83%)	
Poorly/undifferentiated	25 (32.89%)	24 (31.17%)	
Satellite nodule			0.773
No	52 (68.42%)	51 (66.23%)	
Yes	24 (31.58%)	26 (33.77%)	
Cirrhosis			0.028
No	38 (50.00%)	25 (32.47%)	
Yes	38 (50.00%)	52 (67.53%)	
Resection type			0.168
Minor	36 (47.37%)	28 (36.36%)	
Major	40 (52.63%)	49 (63.64%)	
MIS			0.495*
No	71 (93.42%)	74 (96.10%)	
Yes	5 (6.58%)	3 (3.90%)	
Tumor size			0.292
≤5 cm	34 (44.74%)	28 (36.36%)	
>5 cm	42 (55.26%)	49 (63.64%)	
Tumor number			0.169
Single	42 (55.26%)	34 (44.16%)	
Multiple	34 (44.74%)	43 (55.84%)	
VI			0.027
No	49 (64.47%)	36 (46.75%)	
Yes	27 (35.53%)	41 (53.25%)	
Capsule involvement			0.459
No	28 (36.84%)	24 (31.17%)	
Yes	48 (63.16%)	53 (68.83%)	
Margin status			0.746*
R0	72 (94.74%)	72 (93.51%)	
R1	4 (5.26%)	5 (6.49%)	
LN positive			0.669
No	66 (86.84%)	65 (84.42%)	
Yes	10 (13.16%)	12 (15.58%)	
HBV infection			0.057
No	12 (15.79%)	22 (28.57%)	
Yes	64 (84.21%)	55 (71.43%)	
HCV infection			0.245*
No	76 (100.00%)	74 (96.10%)	
Yes	0 (0.00%)	3 (3.90%)	
AFP (ng/ml)			0.075
Normal(≤9)	30 (39.47%)	20 (25.97%)	
Elevated(>9)	46 (60.53%)	57 (74.03%)	
CEA (ng/ml)			0.196
Normal(≤5)	50 (65.79%)	58 (75.32%)	
Elevated(>5)	26 (34.21%)	19 (24.68%)	
CA19‐9 (U/ml)			0.125
Normal(≤37)	39 (51.32%)	30 (38.96%)	
Elevated(>37)	37 (48.68%)	47 (61.04%)	
Anatomic resection			0.689
No	37 (48.68%)	35 (45.45%)	
Yes	39 (51.32%)	42 (54.55%)	
NASH			0.367*
No	75 (98.68%)	73 (94.81%)	
Yes	1 (1.32%)	4 (5.19%)	
Alcoholic hepatitis			0.120*
No	76 (100.00%)	73 (94.81%)	
Yes	0 (0.00%)	4 (5.19%)	
Child–Pugh			0.789
A	65 (85.53%)	67 (87.01%)	
B	11 (14.47%)	10 (12.99%)	
BMI category, kg/m^2^			<0.001*
Underweight	1 (1.32%)	10 (12.99%)	
Normal	35 (46.05%)	51 (66.23%)	
Overweight	34 (44.74%)	16 (20.78%)	
Obese	6 (7.89%)	0 (0.00%)	
BMI, kg/m^2^	24.27 (21.93–25.62)	21.64 (19.73–23.78)	<0.001
HCC‐TNM 8th stage			0.044
I	16 (21.05%)	5 (6.49%)	
II	6 (7.89%)	12 (15.58%)	
III	44 (57.89%)	48 (62.34%)	
IV	10 (13.16%)	12 (15.58%)	
ICC‐TNM 8th stage			0.163
I	15 (19.74%)	7 (9.09%)	
II	10 (13.16%)	13 (16.88%)	
III	51 (67.11%)	57 (74.03%)	
BCLC stage			0.268
A	41 (53.95%)	32 (41.56%)	
B	20 (26.32%)	23 (29.87%)	
C	15 (19.74%)	22 (28.57%)	

Sarcopenia was defined by medians of sex‐specific psoas muscle index (PMI).

Abbreviations: AFP, alpha‐fetoprotein; BCLC stage, Barcelona Clinic Liver Cancer stage; BMI, body mass index; CA19‐9, carbohydrate antigen 19‐9; CEA, carcinoembryonic antigen; HCC‐TNM 8th stage, tumor–node–metastasis stage of 8th edition for hepatocellular carcinoma; ICC‐TNM 8th stage, tumor–node–metastasis stage of 8th edition for intrahepatic cholangiocarcinoma; LN, lymph node; MIS, minimally invasive surgery; NASH, nonalcoholic steatohepatitis; PMI, psoas muscle index; PS, performance status; VI, vascular invasion.

* Fisher's exact probability method.

### 3.2 Long‐term prognosis of the entire study cohort

A total of 109 (71.2%) patients died and 44 (28.6%) survived to the last follow‐up date. The median follow‐up duration was 41.3 months (IQR: 36.2–59.9). The median OS was 17.0 (95% CI: 12.8–21.2) months and the 1‐, 3‐, and 5‐year OS rates were 60.21%, 27.84%, and 21.41%, respectively. The median DFS was 6.7 (95% CI: 4.9–8.4) months and the 1‐, 3‐, and 5‐year DFS rates were 31.39%, 16.99%, and 12.08%, respectively.

### 3.3 Impact of sarcopenia on overall survival (OS) and disease‐free survival (DFS)

**FIGURE 1:**
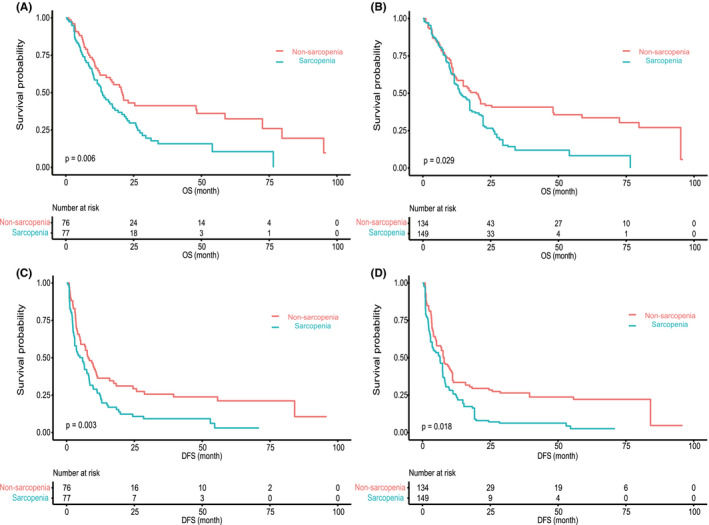
Impact of sarcopenia on overall survival (OS) and disease‐free survival (DFS). (A, C) The K–M curves showed OS and DFS of cHCC‐CC patients after surgery grouped by sarcopenia. (B, D) The K–M curves adjusted all baseline variables using inverse probability weighting (IPW) showed OS and DFS of cHCC‐CC patients after surgery grouped by sarcopenia. DFS, disease‐free survival; OS, overall survival

Significant differences in OS and DFS were found between the non‐sarcopenia and sarcopenia groups using the K–M curves (*p* = 0.006 for OS; *p* = 0.003 for DFS) and the K–M curve adjusted potential confounders with IPW (*p* = 0.029 for OS; *p* = 0.018 for DFS) (Figure 1, Table S3). In summary, the sarcopenia group had a worse prognosis, with a median OS of 13.4 (95% CI: 10.3–19.3) and the 1‐, 3‐, and 5‐year OS rates of 55.84%, 15.80%, and 10.54%, respectively. The non‐sarcopenia group had a median OS of 20.9 (95% CI: 14.9–58.7) and the 1‐, 3‐, and 5‐year OS rates were 64.73%, 41.24%, and 32.48%, respectively. Similarly, the median DFS and 1‐, 3‐, and 5‐year DFS rates of the sarcopenia and non‐sarcopenia groups were 5.9 (95% CI: 3.0–8.2) and 8.0 (95% CI: 5.2–11.4) and 26.37%, 9.25%, and 3.08% and 36.36%, 25.66%, and 21.18%, respectively (Table S3).

### 3.4 Independent prognostic factors in the cHCC‐CC cohort after surgery

Univariable Cox analysis revealed sarcopenia, the satellite nodule status, VI, R1 resection, a relatively large tumor size (>5 cm), multiple tumors, LN involvement, elevated AFP, and CEA as significant risk prognostic factors for OS (*p* < 0.05). Further multivariable analysis after backward selection based on AIC showed that sarcopenia (HR = 1.55; 95% CI: 1.02–2.36; *p* = 0.040), multiple tumors (HR = 1.85; 95% CI: 1.25–2.73; *p* = 0.002), VI (HR = 1.78; 95% CI: 1.19–2.64; *p* = 0.005), an R1 margin (HR = 2.27; 95% CI: 1.07–4.82; *p* = 0.033), LN involvement (HR = 3.25; 95% CI: 1.87–5.65; *p* < 0.001), and elevated CEA (HR = 1.47; 95% CI: 0.94–2.29; *p* = 0.095) were independent prognostic factors (Table 2).

**TABLE 2:** Prognostic factors for cHCC‐CC patients by univariate and multivariate analyses based on OS and DFS

Variables	Univariate analysis (OS)		Multivariate analysis (OS)		Univariate analysis (DFS)		Multivariate analysis (DFS)	
	HR (95% CI)	*p* value	HR (95% CI)	*p* value	HR (95% CI)	*p* value	HR (95% CI)	*p* value
Sarcopenia (Yes/No)	1.72 (1.17, 2.55)	0.006	1.55 (1.02, 2.36)	0.040	1.70 (1.19, 2.43)	0.004	1.55 (1.08, 2.22)	0.019
Age (>55/≤55 years)	1.21 (0.83, 1.76)	0.329*			0.93 (0.65, 1.32)	0.691*		
Gender (Female/Male)	0.64 (0.37, 1.13)	0.123*			0.84 (0.52, 1.34)	0.455*		
PS (0/1–2)	1.11 (0.71, 1.73)	0.662			0.91 (0.60, 1.39)	0.675		
Differentiation (Low/Undiff‐Well/Moder)	1.10 (0.74, 1.64)	0.646*			1.15 (0.79, 1.68)	0.463*		
Satellite (Yes/No)	1.50 (1.01, 2.21)	0.044			1.70 (1.18, 2.45)	0.005		
VI (Yes/No)	1.87 (1.28, 2.74)	0.001	1.78 (1.19, 2.64)	0.005	1.89 (1.33, 2.69)	0.000	1.53 (1.04, 2.25)	0.030
LN metastases (Yes/No)	2.96 (1.80, 4.88)	<0.001	3.25 (1.87, 5.65)	<0.001	2.03 (1.25, 3.29)	0.004	2.04 (1.22, 3.43)	0.007
Capsule involvement (Yes/No)	1.33 (0.89, 1.98)	0.166			1.38 (0.95, 2.00)	0.088		
Margin status (R1/R0)	2.72 (1.32, 5.62)	0.007	2.27 (1.07, 4.82)	0.033	2.46 (1.14, 5.32)	0.022	2.50 (1.13, 5.53)	0.023
Resection type (Major/minor)	1.20 (0.81, 1.76)	0.362			1.57 (1.09, 2.26)	0.015		
Minimal invasive surgery (Yes/No)	0.51 (0.16, 1.63)	0.258			0.68 (0.30, 1.55)	0.358		
Tumor size(>5/≤5 cm)	1.94 (1.30, 2.88)	0.001			2.62 (1.80, 3.82)	<0.001	1.89 (1.25, 2.85)	0.003
Tumor number (Multiple/Single)	2.00 (1.36, 2.94)	<0.001	1.85 (1.25, 2.73)	0.002	2.01 (1.41, 2.87)	0.000	1.88 (1.30, 2.71)	0.001
Anatomic resection (Yes/No)	1.04 (0.71, 1.52)	0.837			1.23 (0.87, 1.75)	0.244		
Cirrhosis (Yes/No)	1.19 (0.81, 1.74)	0.384			1.11 (0.78, 1.59)	0.557		
HBV infection (Yes/No)	1.44 (0.89, 2.32)	0.139			1.37 (0.89, 2.10)	0.156		
HCV infection (Yes/No)	1.08 (0.34, 3.41)	0.894			0.82 (0.26, 2.60)	0.742		
Child–Pugh classification(B/A)	1.11 (0.66, 1.87)	0.693			1.30 (0.81, 2.11)	0.277		
NASH (Yes/No)	1.13 (0.42, 3.09)	0.806			1.24 (0.51, 3.06)	0.636		
Alcoholic hepatitis (Yes/No)	1.11 (0.35, 3.51)	0.859			1.67 (0.62, 4.54)	0.312		
AFP (Elevated/Normal)	1.73 (1.13, 2.65)	0.012			1.57 (1.07, 2.29)	0.020		
CEA (Elevated/Normal)	1.66 (1.11, 2.48)	0.013	1.47 (0.94, 2.29)	0.095	1.62 (1.11, 2.36)	0.012		
CA19‐9 (Elevated/Normal)	1.39 (0.95, 2.05)	0.088			1.39 (0.98, 1.99)	0.067		
Underweight	Ref				Ref			
Normal	0.95 (0.45, 1.99)	0.887			1.11 (0.55, 2.21)	0.778		
Overweight	0.98 (0.45, 2.11)	0.952			0.92 (0.44, 1.90)	0.822		
Obesity	0.88 (0.26, 2.93)	0.834			0.84 (0.28, 2.50)	0.751		

Abbreviations: AFP, alpha‐fetoprotein; BMI, body mass index; CA19‐9, carbohydrate antigen 19‐9; CEA, carcinoembryonic antigen; CI, confidence interval; HR, hazard ratio; LN, lymph node; NASH, nonalcoholic steatohepatitis; Poorly/Undiff, poorly to undifferentiated; VI, vascular invasion; Well/Moder, well to moderately differentiated.

* Clinically relevant variables with *p* > 0.1.

Regarding DFS, univariate analysis suggested that sarcopenia, the satellite nodule status, VI, LN involvement, R1 resection, resection type, a relatively large tumor size (>5 cm), multiple tumors, elevated AFP, and CEA were significant risk prognostic factors (*p* < 0.05), and further multivariate analysis confirmed sarcopenia (HR = 1.55; 95% CI: 1.08–2.22; *p* = 0.019), VI (HR = 1.53; 95% CI: 1.04–2.25; *p* = 0.030), LN involvement (HR = 2.04; 95% CI: 1.22–3.43; *p* = 0.007), an R1 margin (HR = 2.50; 95% CI: 1.13–5.53; *p* = 0.023), multiple tumors (HR = 1.88; 95% CI: 1.30–2.71; *p* = 0.001), and a large tumor size (HR = 1.89; 95% CI: 1.25–2.85; *p* = 0.003) as independent prognostic indicators (Table 2).

### 3.5 Subgroup analysis of the impact of sarcopenia on OS and DFS

**FIGURE 2:**
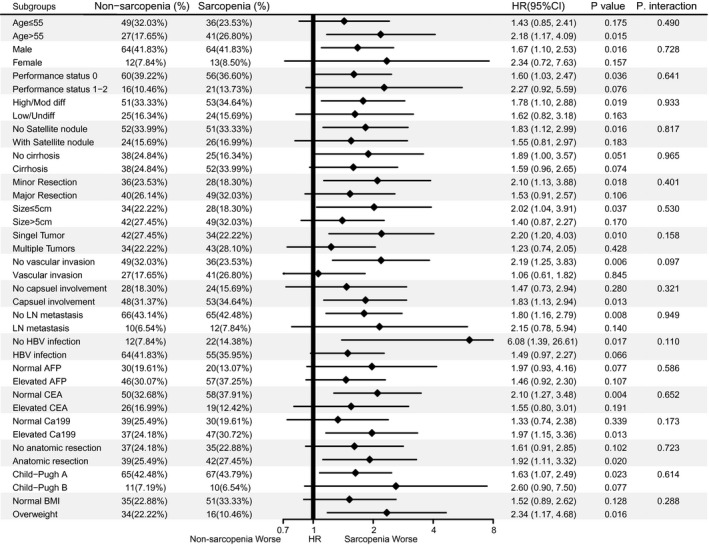
Subgroup analysis and test for interaction to evaluate the impact of sarcopenia on overall survival in cHCC‐CC patients after surgery. AFP, alpha‐fetoprotein; CA19‐9, carbohydrate antigen 19‐9; CEA, carcinoembryonic antigen; HR, hazard ratio; LN, lymph node; OS, overall survival; *p*. inter, *p* value for interaction; Poorly/Undiff, low to undifferentiated; Well/Moder, well to moderately differentiated. In the category of BMI, the underweight group (*n* = 11, 7.19%) and the obese group (*n* = 6, 3.92%) are removed from subgroup analysis for small numbers

**FIGURE 3:**
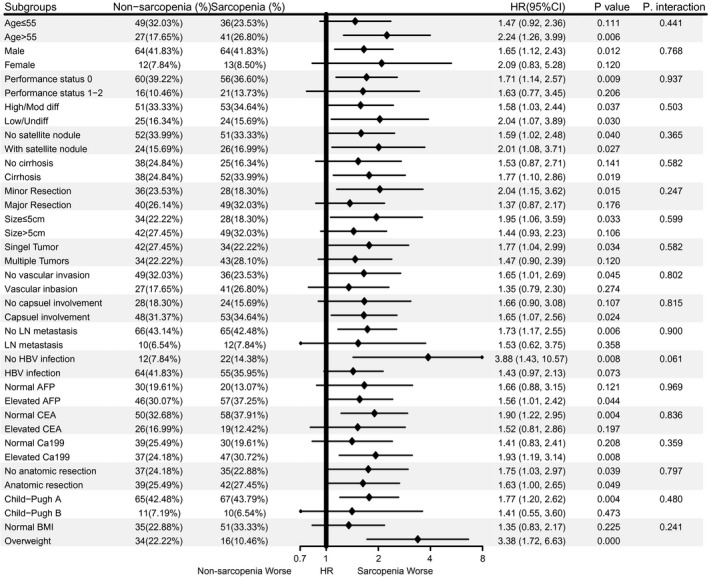
Subgroup analysis and test for interaction to evaluate the impact of sarcopenia on disease‐free survival in cHCC‐CC patients after surgery. AFP, alpha‐fetoprotein; CA19‐9, carbohydrate antigen 19‐9; CEA, carcinoembryonic antigen; DFS, disease‐free survival; HR, hazard ratio; LN, lymph node; *p*. inter, *p* value for interaction; Poorly/Undiff, low to undifferentiated; Well/Moder, well to moderately differentiated. In the category of BMI, the underweight group (*n* = 11, 7.19%) and the obese group (*n* = 6, 3.92%) are removed from subgroup analysis for small numbers

Subgroup analysis according to potential confounders and all baseline variables based on OS (Figure 2) and DFS (Figure 3) suggested that sarcopenia was a risk factor for the prognosis, although no statistical significance was found in some subgroups, and their interactions were not significant. Collectively, sarcopenia is a stable hazard for the prognosis in cHCC‐CC patients after surgery (Tables S4 and S5).

### 3.6 Establishment of a prognostic nomogram for cHCC‐CC patients after surgery

**FIGURE 4:**
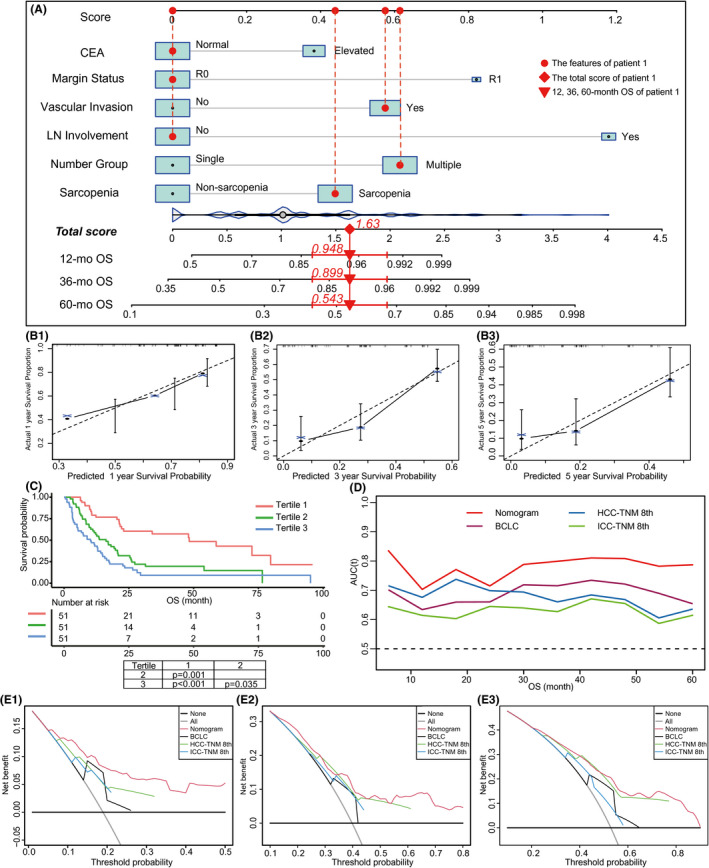
Establishment and evaluation of the prognostic nomogram. (A) Nomogram based on OS for cHCC‐CC patients after surgery. Points are assigned for all risk factors, first by drawing a line upward from the corresponding value to the “Score” line to get the points for each factor, then the points for all factors are added to obtain the total score and a vertical line is drawn to the “Total score” row to determine 1‐, 3‐, and 5‐year survival rates. Patient 1 from this study is shown as an example (presented in red). The distinct area of rectangles represents the difference in the relative proportion of patients in each subgroup. The distribution of total scores is also shown. CEA, carcinoembryonic antigen; mo, month; OS, overall survival. (B) Calibration plots of prognostic nomogram at 1‐, 3‐, and 5‐year survival prediction. (C) Prognostic stratification of the nomogram. Risk scores from all enrolled patients are calculated according to the nomogram and grouped by the tertiles. The Kaplan–Meier plots are depicted and differences between groups are tested (Holm's method). (D) Time‐dependent ROC of nomogram and PLC stages for predicting OS. AUC, area under the curve; ROC, receiver operating characteristic. (E) Decision curve analysis of nomogram and PLC stages for the overall survival prediction of cHCC‐CC patients after surgery at 6 months (E1), 12 months (E2), and 18 months (E3). The net benefits (*y*‐axis) are calculated for nomogram and PLC stages over full range of probability threshold. Horizontal dark solid lines assume no cases will experience the event; gray solid line lines assume all cases will experience the event. BCLC stage, Barcelona Clinic Liver Cancer stage; HCC‐TNM 8th, tumor–node–metastasis stage of 8th edition for hepatocellular carcinoma; ICC‐TNM 8th, tumor–node–metastasis stage of 8th edition for intrahepatic cholangiocarcinoma

To guide the selection of surgical candidates and evaluate the prognosis of cHCC‐CC patients after curative resection, a prognostic nomogram based on the aforementioned multivariate Cox regression was established (Figure 4A). The nomogram recorded a c‐index of 0.696 (95% CI: 0.642–0.750), while the AUC values at 1, 3, and 5 years and integrated AUC values were 0.703 (95% CI: 0.618–0.750), 0.799 (95% CI: 0.706–0.892), 0.787 (95% CI: 0.650–0.924), and 0.780, respectively (Table S6). Calibration plots at 1, 3, and 5 years revealed favorable consistency between the predicted and actual survival rates (Figure 4B). All the patients were stratified by tertiles of predictive scores, and the resulting K–M curves revealed significant prognostic differences between any two adjacent groups (Figure 4C). However, BCLC, HCC‐TNM, and ICC‐TNM stages showed inferior efficacy of prognostic stratification (Figure S3). The Td‐ROC curves revealed that our nomogram had higher AUCs than BCLC, HCC‐TNM, and ICC‐TNM stages at any given month within 5‐year post‐surgery (Figure 4D). The NRI and IDI derived from the comparison between our nomogram and PLC stages at 1, 3, and 5 years revealed consistently positive improvement, and most of the *p* values were significant (Table 3). A fivefold cross‐validation consistently suggested superior prediction of nomogram than PLC stage, revealing higher c‐index, AUC, and lower Brier score (Table 4). The DCA curves at 6, 12, and 18 months revealed that applying our nomogram to inform clinical decisions would lead to superior outcomes than BCLC, HCC‐TNM, and ICC‐TNM stages over a wide range of threshold probabilities (Figure 4E). Collectively, our nomogram outperformed other PLC stages in discrimination and clinical application.

**TABLE 3:** NRI and IDI derived from the comparison between nomogram and PLC stages for cHCC‐CC patients after surgery

Variables	1 year	*p* value	3 years	*p* value	5 years	*p* value
	Estimate (95% CI)		Estimate (95% CI)		Estimate (95% CI)	
Nomo‐ICC TNM 8th						
IDI	14.2% (6.9%, 23.6%)	<0.001	14.9% (4.7%, 27.8%)	0.010	13.7% (1.1%, 27.7%)	0.030
NRI	20.0% (1.0%, 44.5%)	0.030	36.6% (2.0%, 57.4%)	0.020	45.1% (−5.6%, 64.7%)	0.119
Nomo‐HCC TNM 8th						
IDI	8.8% (3.8%, 17.4%)	0.010	11.3% (0.8%, 22.0%)	0.040	11.6% (−1.0%, 24.7%)	0.090
NRI	17.1% (−1.9%, 41.1%)	0.109	29.3% (−8.6%, 53.7%)	0.119	35.3% (−3.4%, 61.6%)	0.070
Nomo‐BCLC						
IDI	15.8% (8.2%, 25.5%)	<0.001	15.0% (3.3%, 25.7%)	0.010	14.7% (2.1%, 29.2%)	0.020
NRI	37.1% (12.8%, 51.1%)	<0.001	39.1% (14.3%, 55.9%)	0.010	39.4% (11.3%, 66.9%)	0.020

Abbreviations: BCLC stage, Barcelona Clinic Liver Cancer stage; HCC‐TNM 8th, tumor–node–metastasis stage of 8th edition for hepatocellular carcinoma; ICC‐TNM 8th, tumor–node–metastasis stage of 8th edition for intrahepatic cholangiocarcinoma; IDI, integrated discrimination improvement; NRI, net reclassification index; PLC, primary liver cancer.

**TABLE 4:** The comparison between nomogram and PLC stages in prognostic prediction by a fivefold cross‐validation

Models	1 year			3 years			5 years		
	C‐index	AUC	Brier score	C‐index	AUC	Brier score	C‐index	AUC	Brier score
Nomogram	0.686	0.681	0.219	0.686	0.831	0.174	0.686	0.791	0.155
HCC TNM 8th	0.645	0.682	0.228	0.645	0.680	0.199	0.645	0.635	0.176
ICC TNM 8th	0.589	0.620	0.235	0.589	0.650	0.195	0.589	0.614	0.173
BCLC stage	0.614	0.625	0.230	0.614	0.749	0.183	0.614	0.686	0.159

Abbreviations: BCLC stage, Barcelona Clinic Liver Cancer stage; HCC‐TNM 8th, tumor–node–metastasis stage of 8th edition for hepatocellular carcinoma; ICC‐TNM 8th, tumor–node–metastasis stage of 8th edition for intrahepatic cholangiocarcinoma.

## 4 DISCUSSION

This retrospective study revealed for the first time that sarcopenia is a stable and independent prognostic indicator for OS (HR = 1.55; 95% CI: 1.02–2.36; *p* = 0.040) and DFS (HR = 1.55; 95% CI: 1.08–2.22; *p* = 0.019) in cHCC‐CC patients after surgery. This finding is consistent with previous studies, indicating that sarcopenia is related to a poor prognosis in HCC and ICC patients after surgery.^16^, ^17^ The nomogram based on sarcopenia outperforms the available PLC stage in survival prediction, likely aiding surgical candidate selection and early intervention to improve survival.

Although first described in 1903,^35^ cHCC‐CC remains an uncommon subtype of PLC with scant attention.^1^, ^3^, ^4^ In the context of increasingly standardized management of HCC and ICC, population‐based research unfortunately unraveled a gradual increase in occurrence and mortality in cHCC‐CC patients.^36^ Therefore, comprehensive identification of risk indicators for the prognosis could aid clinical decisions to improve outcomes. Previous studies have reported that multiple tumors, a large tumor size, a resection margin, vascular invasion, satellite nodules, and tumor markers were risk factors for a poor postoperative prognosis in cHCC‐CC patients.^9^, ^37^, ^38^, ^39^ Although sarcopenia was proved to be an independent risk factor for a poor prognosis in various malignancies, however, its effect on the prognosis of cHCC‐CC remains unclear.

Sarcopenia originally refers to a loss of skeletal muscle mass and function with age and chronic diseases.^40^ Currently, a consensus concerning the definition of sarcopenia has yet to be reached for progress and updates in content.^41^ According to the European Working Group definition on Sarcopenia in Older People (EWGSOP) and updates in 2019, the diagnosis should consider muscle strength, muscle mass, or quality.^42^, ^43^ Because of the inconvenience of quantifying muscle function in clinical assessments, the skeletal muscle area at the level of L3 based on CT was chosen in most studies as an appropriate method to evaluate muscle mass.^23^, ^24^ Although other body composition indexes, such as muscle attenuation (MA), visceral adipose tissue index, and subcutaneous adipose tissue index, independently predict the prognosis in cancer patients,^44^, ^45^ the requirement of sophisticated calculation and complex measurement limits its clinical application. Therefore, we chose the PMI to evaluate sarcopenia in our study because it is easy to measure in a clinical setting. Moreover, although a transformation of continuous PMI to binary variable is possibly arbitrary and can limit statistical power, but it is conceptually convenient and straightforward for clinical use. Considering the lack of an available reference PMI in cHCC‐CC patients, we defined the sex‐specific median as the cut‐off value to explore the cHCC‐CC‐specific assessment for sarcopenia. In our study, the cut‐off values of the PMI for male and female individuals were 5.42 and 4.05 cm^2^/m^2^, respectively, which were comparable to previous studies in Asia.^46^, ^47^

Although cHCC‐CC was incorporated into the ICC‐TNM stage in 2010,^7^ an increasing number of studies have revealed low prognostic efficiency.^9^, ^10^, ^11^ Usually, conventional tumor stages focus on tumor characteristics and liver function; however, the general state is also vital for the prognosis of cancer patients. Although performance status is an important variable in the BCLC stage,^8^ patients with PS scores of 3–4 were routinely excluded from surgery, limiting its prognostic efficiency in cancer patients after surgery. Consistent with previous studies in many cancers,^12^, ^13^, ^14^, ^15^, ^16^, ^17^ univariate and multivariable Cox analyses in our study revealed that sarcopenia is an independent poor prognosis factor for OS and DFS in cHCC‐CC patients undergoing surgical treatment. The sarcopenia group revealed a worse prognosis, probably resulting from a more common malnutritional status and a decrease in immune function,^48^ and the molecular mechanisms require further investigation. Further subgroup analysis of potential confounders and all baseline variables confirmed that sarcopenia is a stable predictor of a poor prognosis. Additionally, PMI could be calculated easily based on routine preoperative abdominal CT without adding costs and consuming time. Collectively, our study confirmed the impact of sarcopenia on cHCC‐CC patients after surgery and underscored the necessity to incorporate PMI and sarcopenia into prognostic prediction.

Prognostic models could be evaluated by indexes of discrimination, consistency, and clinical validity.^49^ The c‐index and AUCs have been widely used in discrimination. Our study used td‐ROC for its superiority over conventional ROC for survival data.^28^ Our nomogram had a higher c‐index and higher AUCs of td‐ROC within 60 months after surgery, similar to the integrated AUC. NRI is increasingly used to quantify the improvement in the reclassification of a new model over the original model,^29^, ^30^ while the IDI is accumulatively applied to evaluate the overall improvement of a predictive model.^30^ Both the NRI and IDI revealed positive improvement, indicating better predictive accuracy than PLC stages. Additionally, the calibration curves revealed favorable consistency between the predictions and actual observations. DCA was used to quantify the net benefits at different threshold probabilities to evaluate the clinical usefulness of the predictive model, further showing that the application of our nomogram to inform clinical decisions would lead to superior outcomes than PLC stages over a wide range of threshold probabilities.^31^, ^50^ Additionally, our nomogram, but not PLC stages, stratified patients into groups with significantly different prognoses. Reliable stratification could aid high‐risk patient identification for further effective interventions, including exercise intervention,^51^ nutrition intervention,^52^ and pharmacological interventions.^53^ Overall, our nomogram outperforms PLC stages in discrimination, calibration, and clinical effectiveness.

Our study has several limitations. First, this study was a single‐center retrospective study with limited cases due to the low incidence and difficult diagnosis of cHCC‐CC. However, the sample size was comparable to or larger than previous studies as a single‐center study.^9^, ^10^, ^11^ Second, sarcopenia was defined by the PMI, which varied because of geographic heterogenicity. Although whether the cut‐off values of the PMI can be applied to patients in Western countries requires further investigation, the adverse effect of sarcopenia on prognosis is convincible. Third, our nomogram has only been internally validated thus far. Although internal validation is considered a prerequisite for prediction model development when data are limited^54^ and the internal validation based on resampling technique and cross‐validation revealed favorably consistent results, external validation is still required for generalization. Therefore, further prospective and multicenter studies with large‐scale patients are required to validate our findings.

## 5 CONCLUSIONS

We found that sarcopenia, defined by the PMI based on preoperative CT scans, was an independent and stable adverse prognostic factor in cHCC‐CC patients after surgery. Our nomogram based on sarcopenia and clinicopathological characteristics reveals superior prognostic efficacy over PLC stages, which may aid high‐risk patient identification and clinical decisions. Corresponding early intervention is expected to improve the prognosis of cHCC‐CC sarcopenia patients after surgery.

## CONFLICT OF INTEREST

The authors declare no conflict of interest.

## AUTHORS’ CONTRIBUTIONS

Conceptualization: Gui‐min Hou and Chuang Jiang; Methodology: Gui‐min Hou, Chuang Jiang, and Jin‐peng Du; Statistical analysis: Gui‐min Hou, Chuang Jiang, and Ke‐fei Yuan; Investigation: Ke‐fei Yuan; Writing: Gui‐min Hou and Chuang Jiang; Supervision, Ke‐fei Yuan. All authors have read and approved the final manuscript.

## ETHICAL APPROVAL STATEMENT

This study was approved by the ethics committee of West China Hospital.

## Supporting information

Figure S1

Figure S2

Figure S3

Tables S1–S6

## Data Availability

All data included in this study are available upon request by contact with the corresponding author.
