# Supplementary material for: Sarcopenia predicts an adverse prognosis in patients with combined hepatocellular carcinoma and cholangiocarcinoma after surgery
Source: Cancer Med. 2021 Dec 5;11(2):317–31. doi: 10.1002/cam4.4448 (PMC8729053; doi:10.1002/cam4.4448)
Supplement: Supplementary file 4 — Tables S1–S6 [file CAM4-11-317-s001.docx]

**Supplementary materials**

| Table s1: The comparison between the enrolled patients and the excluded patients for absence of PMI | | | |
| --- | --- | --- | --- |
| Group | Enrolled | Excluded for absence of PMI | P-value |
| N | 153 | 16 |  |
| DFS (Median, Q1-Q3) | 6.60 (2.60-12.90) | 2.75 (1.80-7.73) | 0.118§ |
| OS (Median, Q1-Q3) | 13.60 (7.50-26.10) | 8.25 (2.90-47.70) | 0.422§ |
| Age (years) |  |  | 0.594 |
| ≤55 | 85 (55.56%) | 10 (62.50%) |  |
| >55 | 68 (44.44%) | 6 (37.50%) |  |
| Gender |  |  | 0.173* |
| Male | 122 (79.74%) | 15 (93.75%) |  |
| Female | 31 (20.26%) | 1 (6.25%) |  |
| Performance status |  |  | 0.627* |
| 0 | 116 (75.82%) | 13 (81.25%) |  |
| 1-2 | 37 (24.18%) | 3 (18.75%) |  |
| Differentiation |  |  | 0.343 |
| Well/moderately | 104 (67.97%) | 9 (56.25%) |  |
| Poorly/undifferentiated | 49 (32.03%) | 7 (43.75%) |  |
| Satellite nodule |  |  | 0.06 |
| No | 103 (67.32%) | 7 (43.75%) |  |
| Yes | 50 (32.68%) | 9 (56.25%) |  |
| Cirrhosis |  |  | 0.441 |
| No | 63 (41.18%) | 5 (31.25%) |  |
| Yes | 90 (58.82%) | 11 (68.75%) |  |
| Resection Type |  |  | 0.882 |
| Minor | 64 (41.83%) | 7 (43.75%) |  |
| Major | 89 (58.17%) | 9 (56.25%) |  |
| MIS |  |  | 0.349* |
| No | 145 (94.77%) | 16 (100.00%) |  |
| Yes | 8 (5.23%) | 0 (0.00%) |  |
| Tumor size |  |  | 0.226* |
| ≤5cm | 62 (40.52%) | 4 (25.00%) |  |
| >5cm | 91 (59.48%) | 12 (75.00%) |  |
| Tumor number |  |  | 0.652 |
| Single | 76 (49.67%) | 7 (43.75%) |  |
| Multiple | 77 (50.33%) | 9 (56.25%) |  |
| VI |  |  | 0.594 |
| No | 85 (55.56%) | 10 (62.50%) |  |
| Yes | 68 (44.44%) | 6 (37.50%) |  |
| Capsule involvement |  |  | 0.079* |
| No | 52 (33.99%) | 2 (12.50%) |  |
| Yes | 101 (66.01%) | 14 (87.50%) |  |
| Margin status |  |  | 0.953* |
| R0 | 144 (94.12%) | 15 (93.75%) |  |
| R1 | 9 (5.88%) | 1 (6.25%) |  |
| LN positive |  |  | 0.639* |
| No | 131 (85.62%) | 13 (81.25%) |  |
| Yes | 22 (14.38%) | 3 (18.75%) |  |
| HBV infection |  |  | 0.415 |
| No | 34 (22.22%) | 5 (31.25%) |  |
| Yes | 119 (77.78%) | 11 (68.75%) |  |
| HCV infection |  |  | 0.572* |
| No | 150 (98.04%) | 16 (100.00%) |  |
| Yes | 3 (1.96%) | 0 (0.00%) |  |
| AFP (ng/mL) |  |  | 0.908 |
| Normal(≤9) | 50 (32.68%) | 5 (31.25%) |  |
| Elevated(>9) | 103 (67.32%) | 11 (68.75%) |  |
| CEA (ng/mL) |  |  | 0.711* |
| Normal(≤5) | 108 (70.59%) | 12 (75.00%) |  |
| Elevated(>5) | 45 (29.41%) | 4 (25.00%) |  |
| CA19-9 (U/mL) |  |  | 0.561 |
| Normal(≤37) | 69 (45.10%) | 6 (37.50%) |  |
| Elevated(>37) | 84 (54.90%) | 10 (62.50%) |  |
| Anatomic resection |  |  | 0.099 |
| No | 72 (47.06%) | 11 (68.75%) |  |
| Yes | 81 (52.94%) | 5 (31.25%) |  |
| NASH |  |  | 0.463* |
| No | 148 (96.73%) | 16 (100.00%) |  |
| Yes | 5 (3.27%) | 0 (0.00%) |  |
| Alcoholic hepatitis |  |  | 0.414* |
| No | 149 (97.39%) | 15 (93.75%) |  |
| Yes | 4 (2.61%) | 1 (6.25%) |  |
| Child-Pugh |  |  | 0.113* |
| A | 132 (86.27%) | 16 (100.00%) |  |
| B | 21 (13.73%) | 0 (0.00%) |  |
| HCC-TNM 8th stage |  |  | 0.151* |
| I | 21 (13.73%) | 0 (0.00%) |  |
| II | 18 (11.76%) | 0 (0.00%) |  |
| III | 92 (60.13%) | 13 (81.25%) |  |
| IV | 22 (14.38%) | 3 (18.75%) |  |
| ICC-TNM 8th stage |  |  | 0.230* |
| I | 22 (14.38%) | 0 (0.00%) |  |
| II | 23 (15.03%) | 2 (12.50%) |  |
| III | 108 (70.59%) | 14 (87.50%) |  |
| BCLC stage |  |  | 0.715 |
| A | 73 (47.71%) | 7 (43.75%) |  |
| B | 43 (28.10%) | 6 (37.50%) |  |
| C | 37 (24.18%) | 3 (18.75%) |  |
| **Abbreviations:** PMI: psoas muscle index; Q: quartile; DFS: disease free survival; OS: overall survival; PS: performance status; MIS: Minimally invasive surgery; LN: lymph node; VI: vascular invasion; NASH: nonalcoholic steatohepatitis; AFP: alpha-fetoprotein; CEA: carcinoembryonic antigen; CA19-9: carbohydrate antigen 19-9, BMI: body mass index; BCLC stage: Barcelona Clinic Liver Cancer stage; HCC-TNM 8th: tumor-node-metastasis stage of 8th edition for hepatocellular carcinoma; ICC-TNM 8th: tumor-node-metastasis stage of 8th edition for intrahepatic cholangiocarcinoma; *: Fisher’s exact probability method; §: The Kruskal Wallis rank sum test was used. | | | |
|  |  |  |  |

| Table s2. The baseline characteristics of the 153 cHCC-CC patients | |
| --- | --- |
| Variables | N (%) |
| PMI male, cm^2^/m^2^ § | 5.42 (4.50-7.01) |
| PMI female, cm^2^/m^2^ § | 4.05 (3.44-4.31) |
| Age (years) |  |
| ≤55 | 85 (55.56%) |
| >55 | 68 (44.44%) |
| Gender |  |
| Male | 128 (83.66%) |
| Female | 25 (16.34%) |
| Performance status |  |
| 0 | 116 (75.82%) |
| 1-2 | 37 (24.18%) |
| Differentiation |  |
| Well/moderately | 104 (67.97%) |
| Poorly/undifferentiated | 49 (32.03%) |
| Satellite nodule |  |
| No | 103 (67.32%) |
| Yes | 50 (32.68%) |
| Cirrhosis |  |
| No | 63 (41.18%) |
| Yes | 90 (58.82%) |
| Resection Type |  |
| Minor | 64 (41.83%) |
| Major | 89 (58.17%) |
| MIS |  |
| No | 145 (94.77%) |
| Yes | 8 (5.23%) |
| Tumor size |  |
| ≤5cm | 62 (40.52%) |
| >5cm | 91 (59.48%) |
| Tumor number |  |
| Single | 76 (49.67%) |
| Multiple | 77 (50.33%) |
| VI |  |
| No | 85 (55.56%) |
| Yes | 68 (44.44%) |
| Capsule involvement |  |
| No | 52 (33.99%) |
| Yes | 101 (66.01%) |
| Margin status |  |
| R0 | 144 (94.12%) |
| R1 | 9 (5.88%) |
| LN positive |  |
| No | 131 (85.62%) |
| Yes | 22 (14.38%) |
| HBV infection |  |
| No | 34 (22.22%) |
| Yes | 119 (77.78%) |
| HCV infection |  |
| No | 150 (98.04%) |
| Yes | 3 (1.96%) |
| AFP (ng/mL) |  |
| Normal(≤9) | 50 (32.68%) |
| Elevated(>9) | 103 (67.32%) |
| CEA (ng/mL) |  |
| Normal(≤5) | 108 (70.59%) |
| Elevated(>5) | 45 (29.41%) |
| CA19-9 (U/mL) |  |
| Normal(≤37) | 69 (45.10%) |
| Elevated(>37) | 84 (54.90%) |
| Anatomic resection |  |
| No | 72 (47.06%) |
| Yes | 81 (52.94%) |
| NASH |  |
| No | 148 (96.73%) |
| Yes | 5 (3.27%) |
| Alcoholic hepatitis |  |
| No | 149 (97.39%) |
| Yes | 4 (2.61%) |
| Child-Pugh |  |
| A | 132 (86.27%) |
| B | 21 (13.73%) |
| BMI category, kg/m2 |  |
| Underweight | 11 (7.19%) |
| Normal | 86 (56.21%) |
| Overweight | 50 (32.68%) |
| Obese | 6 (3.92%) |
| HCC-TNM 8th stage |  |
| I | 21 (13.73%) |
| II | 18 (11.76%) |
| III | 92 (60.13%) |
| IV | 22 (14.38%) |
| ICC-TNM 8th stage |  |
| I | 22 (14.38%) |
| II | 23 (15.03%) |
| III | 108 (70.59%) |
| BCLC stage |  |
| A | 73 (47.71%) |
| B | 43 (28.10%) |
| C | 37 (24.18%) |
| Abbreviations: PMI: psoas muscle index; PS: performance status; MIS: Minimally invasive surgery; LN: lymph node; VI: vascular invasion; NASH: nonalcoholic steatohepatitis; AFP: alpha-fetoprotein; CEA: carcinoembryonic antigen; CA19-9: carbohydrate antigen 19-9, BMI: body mass index; BCLC stage: Barcelona Clinic Liver Cancer stage; HCC-TNM 8th: tumor-node-metastasis stage of 8th edition for hepatocellular carcinoma; ICC-TNM 8th: tumor-node-metastasis stage of 8th edition for intrahepatic cholangiocarcinoma; §: median (Q1-Q3). | |

| Table s3. The prognosis for the non-sarcopenia and sarcopenia group. | | | | | | |
| --- | --- | --- | --- | --- | --- | --- |
| Variables | Group | Median, 95%CI | 1-y survival rate, 95%CI | 3-y survival rate, 95%CI | 5-y survival rate, 95%CI | p value |
| OS | non-sarcopenia | 20.9 (14.9, 58.7) | 64.73% (54.64%, 76.69%) | 41.24% (30.73%, 55.35%) | 32.48% (21.67%, 48.67%) | 0.006 |
|  | sarcopenia | 13.4 (10.3, 19.3) | 55.84% (45.78%, 68.11%) | 15.80% (8.93%, 27.99%) | 10.54% (3.94%, 28.17%) |  |
| DFS | non-sarcopenia | 8.0 (5.2, 11.4) | 36.36% (26.77%, 49.37%) | 25.66% (16.94%, 38.88%) | 21.18% (12.89%, 34.82%) | 0.003 |
|  | sarcopenia | 5.9 (3.0, 8.2) | 26.37% (18.11%, 38.40%) | 9.25% (4.43%, 19.31%) | 3.08% (0.53%, 17.94%) |  |
| **Abbreviations:** OS: overall survival; DFS: disease free survival. | | | | | | |
|  |  |  |  |  |  |  |

| Table s4. Subgroup analysis according to all baseline characteristics based on OS. | | | | |
| --- | --- | --- | --- | --- |
| Subgroups | N (total) | HR (95%CI) | p value | p. interaction |
| Age≤55 | 85 | 1.43 (0.85, 2.41) | 0.175 | 0.490 |
| Age>55 | 68 | 2.18 (1.17, 4.09) | 0.015 |  |
| Male | 128 | 1.67 (1.10, 2.53) | 0.016 | 0.728 |
| Female | 25 | 2.34 (0.72, 7.63) | 0.157 |  |
| PS 0 | 116 | 1.60 (1.03, 2.47) | 0.036 | 0.641 |
| PS 1-2 | 37 | 2.27 (0.92, 5.59) | 0.076 |  |
| High/Mod diff | 104 | 1.78 (1.10, 2.88) | 0.019 | 0.933 |
| Low/Undiff | 49 | 1.62 (0.82, 3.18) | 0.163 |  |
| No Satellite nodule | 103 | 1.83 (1.12, 2.99) | 0.016 | 0.817 |
| With Satellite nodule | 50 | 1.55 (0.81, 2.97) | 0.183 |  |
| No cirrhosis | 63 | 1.89 (1.00, 3.57) | 0.051 | 0.965 |
| Cirrhosis | 90 | 1.59 (0.96, 2.65) | 0.074 |  |
| Minor Resection | 64 | 2.10 (1.13, 3.88) | 0.018 | 0.401 |
| Major Resection | 89 | 1.53 (0.91, 2.57) | 0.106 |  |
| No MIS | 145 | 1.77 (1.19, 2.64) | 0.005 | NA |
| MIS | 8 | NA |  |  |
| Size≤5cm | 62 | 2.02 (1.04, 3.91) | 0.037 | 0.530 |
| Size>5cm | 91 | 1.40 (0.87, 2.27) | 0.170 |  |
| Single Tumor | 76 | 2.20 (1.20, 4.03) | 0.010 | 0.158 |
| Multiple Tumors | 77 | 1.23 (0.74, 2.05) | 0.428 |  |
| No VI | 85 | 2.19 (1.25, 3.83) | 0.006 | 0.097 |
| VI | 68 | 1.06 (0.61, 1.82) | 0.845 |  |
| No capsule involvement | 52 | 1.47 (0.73, 2.94) | 0.280 | 0.321 |
| Capsule involvement | 101 | 1.83 (1.13, 2.94) | 0.013 |  |
| R0 Resection | 144 | 1.78 (1.19, 2.67) | 0.005 | NA |
| R1 Resection | 9 | NA |  |  |
| N0 | 131 | 1.80 (1.16, 2.79) | 0.008 | 0.949 |
| N1 | 22 | 2.15 (0.78, 5.94) | 0.140 |  |
| No HBV | 34 | 6.08 (1.39, 26.61) | 0.017 | 0.110 |
| HBV | 119 | 1.49 (0.97, 2.27) | 0.066 |  |
| No HCV | 150 | 1.73 (1.17, 2.57) | 0.006 | NA |
| HCV | 3 | NA |  |  |
| Normal AFP | 50 | 1.97 (0.93, 4.16) | 0.077 | 0.586 |
| Elevated AFP | 103 | 1.46 (0.92, 2.30) | 0.107 |  |
| Normal CEA | 108 | 2.10 (1.27, 3.48) | 0.004 | 0.652 |
| Elevated CEA | 45 | 1.55 (0.80, 3.01) | 0.191 |  |
| Normal Ca199 | 69 | 1.33 (0.74, 2.38) | 0.339 | 0.173 |
| Elevated Ca199 | 84 | 1.97 (1.15, 3.36) | 0.013 |  |
| No Anatomic | 72 | 1.61 (0.91, 2.85) | 0.102 | 0.723 |
| Anatomic | 81 | 1.92 (1.11, 3.32) | 0.020 |  |
| No NASH | 148 | 1.77 (1.19, 2.63) | 0.005 | NA |
| NASH | 5 | NA |  |  |
| No AH | 149 | 1.74 (1.17, 2.58) | 0.006 | NA |
| AH | 4 | NA |  |  |
| Child-Pugh A | 132 | 1.63 (1.07, 2.49) | 0.023 | 0.614 |
| Child-Pugh B | 21 | 2.60 (0.90, 7.50) | 0.077 |  |
| Underweight | 11 | NA |  | NA |
| Normal | 86 | 1.52 (0.89, 2.62) | 0.128 |  |
| Overweight | 50 | 2.34 (1.17, 4.68) | 0.016 |  |
| Obese | 6 | NA |  |  |
| **Abbreviations:** HR: hazard ratio; CI: confidence interval; p. inter: p value for interaction. PS: performance status; MIS: Minimally invasive surgery; LN: lymph node; VI: vascular invasion; NASH: nonalcoholic steatohepatitis; AFP: alpha-fetoprotein; CEA: carcinoembryonic antigen; CA19-9: carbohydrate antigen 19-9, BMI: body mass index. | | | | |
|  |  |  |  |  |

| Table s5. Subgroup analysis according to all baseline characteristics based on DFS. | | | | |
| --- | --- | --- | --- | --- |
| Subgroups | N (total) | HR (95%CI) | P value | P. interaction |
| Age≤55 | 85 | 1.47 (0.92, 2.36) | 0.111 | 0.441 |
| Age>55 | 68 | 2.24 (1.26, 3.99) | 0.006 |  |
| Male | 128 | 1.65 (1.12, 2.43) | 0.012 | 0.768 |
| Female | 25 | 2.09 (0.83, 5.28) | 0.120 |  |
| PS 0 | 116 | 1.71 (1.14, 2.57) | 0.009 | 0.937 |
| PS 1-2 | 37 | 1.63 (0.77, 3.45) | 0.206 |  |
| High/Mod diff | 104 | 1.58 (1.03, 2.44) | 0.037 | 0.503 |
| Low/Undiff | 49 | 2.04 (1.07, 3.89) | 0.030 |  |
| No Satellite | 103 | 1.59 (1.02, 2.48) | 0.040 | 0.365 |
| With Satellite | 50 | 2.01 (1.08, 3.71) | 0.027 |  |
| No cirrhosis | 63 | 1.53 (0.87, 2.71) | 0.141 | 0.582 |
| Cirrhosis | 90 | 1.77 (1.10, 2.86) | 0.019 |  |
| Minor Resection | 64 | 2.04 (1.15, 3.62) | 0.015 | 0.247 |
| Major Resection | 89 | 1.37 (0.87, 2.17) | 0.176 |  |
| No MIS | 145 | 1.68 (1.16, 2.42) | 0.006 | NA |
| MIS | 8 | NA |  |  |
| Size≤5cm | 62 | 1.95 (1.06, 3.59) | 0.033 | 0.599 |
| Size>5cm | 91 | 1.44 (0.93, 2.23) | 0.106 |  |
| Single Tumor | 76 | 1.77 (1.04, 2.99) | 0.034 | 0.582 |
| Multiple Tumors | 77 | 1.47 (0.90, 2.39) | 0.120 |  |
| No VI | 85 | 1.65 (1.01, 2.69) | 0.045 | 0.802 |
| VI | 68 | 1.35 (0.79, 2.30) | 0.274 |  |
| No capsule involvement | 52 | 1.66 (0.90, 3.08) | 0.107 | 0.815 |
| Capsule involvement | 101 | 1.65 (1.07, 2.56) | 0.024 |  |
| R0 Resection | 144 | 1.71 (1.18, 2.46) | 0.004 | NA |
| R1 Resection | 9 | NA |  |  |
| N0 | 131 | 1.73 (1.17, 2.55) | 0.006 | 0.900 |
| N1 | 22 | 1.53 (0.62, 3.75) | 0.358 |  |
| No HBV | 34 | 3.88 (1.43, 10.57) | 0.008 | 0.061 |
| HBV | 119 | 1.43 (0.97, 2.13) | 0.073 |  |
| No HCV | 150 | 1.74 (1.21, 2.49) | 0.003 | NA |
| HCV | 3 | NA |  |  |
| Normal AFP | 50 | 1.66 (0.88, 3.15) | 0.121 | 0.969 |
| Elevated AFP | 103 | 1.56 (1.01, 2.42) | 0.044 |  |
| Normal CEA | 108 | 1.90 (1.22, 2.95) | 0.004 | 0.836 |
| Elevated CEA | 45 | 1.52 (0.81, 2.86) | 0.197 |  |
| Normal Ca199 | 69 | 1.41 (0.83, 2.41) | 0.208 | 0.359 |
| Elevated Ca199 | 84 | 1.93 (1.19, 3.14) | 0.008 |  |
| No Anatomic | 72 | 1.75 (1.03, 2.97) | 0.039 | 0.797 |
| Anatomic | 81 | 1.63 (1.00, 2.65) | 0.049 |  |
| No NASH | 148 | 1.71 (1.19, 2.46) | 0.004 | NA |
| NASH | 5 | NA |  |  |
| No AH | 149 | 1.67 (1.17, 2.40) | 0.005 | NA |
| AH | 4 | NA |  |  |
| Child-Pugh A | 132 | 1.77 (1.20, 2.62) | 0.004 | 0.480 |
| Child-Pugh B | 21 | 1.41 (0.55, 3.60) | 0.473 |  |
| Underweight | 11 | NA |  | NA |
| Normal | 86 | 1.35 (0.83, 2.17) | 0.225 |  |
| Overweight | 50 | 3.38 (1.72, 6.63) | 0.000 |  |
| Obesity | 6 | NA |  |  |
| **Abbreviations:** HR: hazard ratio; CI: confidence interval; p. inter: p value for interaction. Well/Moder: well to moderately differentiated; Poorly/Undiff: poorly to undifferentiated. LN: lymph node; VI: vascular invasion; NASH: non-alcoholic steatohepatitis. | | | | |
|  |  |  |  |  |

| Table s6. Comparison between nomogram and PLC stages in prognostic prediction for cHCC-CC patients after surgery. | | | | | |
| --- | --- | --- | --- | --- | --- |
|  | C-index(95%CI) | AUC (95%CI) of 1-year | AUC (95%CI) of 3-year | AUC (95%CI) of 5-year | integrate AUC |
| Nomogram | 0.696 (0.642, 0.750) | 0.703 (0.618, 0.788) | 0.799 (0.706, 0.892) | 0.787 (0.650, 0.924) | 0.780 |
| BCLC stage | 0.618 (0.566, 0.671) | 0.634 (0.548, 0.720) | 0.715 (0.626, 0.805) | 0.654 (0.509, 0.799) | 0.689 |
| HCC-TNM 8th | 0.639 (0.591, 0.687) | 0.676 (0.600, 0.752) | 0.660 (0.559, 0.761) | 0.636 (0.494, 0.777) | 0.678 |
| ICC-TNM 8th | 0.584 (0.539, 0.628) | 0.614 (0.543, 0.685) | 0.627 (0.515, 0.739) | 0.615 (0.443, 0.786) | 0.630 |
| **Abbreviations**: PLC: primary liver cancer; AUC: area under the curve; BCLC stage: Barcelona Clinic Liver Cancer stage; HCC-TNM 8th: tumor-node-metastasis stage of 8th edition for hepatocellular carcinoma; ICC-TNM 8th: tumor-node-metastasis stage of 8th edition for intrahepatic cholangiocarcinoma. | | | | | |
|  |  |  |  |  |  |

**Figure legends**

**Figure s1. Flowchart of all included and excluded patients.**

**Figure s2. The measurement of Psoas muscle area (PMA).**

Psoas muscle area (PMA) was measured at the level of L3 in axial imaging with both vertebral spines visible. Measurement was performed with manual outlining of psoas muscle borders and calculated as follows: PMA =a×b×π，where "a" and "b" are the radii of the major and minor axes, respectively.

**Figure s3. Overall survival of cHCC-CC patients stratified by PLC stages.**

a: The K-M curves showed OS of patients grouped by HCC-TNM 8th stage. b: The K-M curves showed OS of patients grouped by ICC-TNM 8th stage. c: The K-M curves showed OS of patients grouped by BCLC stage. Survival differences between groups were tested (Holm’s method). BCLC: Barcelona Clinic Liver Cancer stage; HCC-TNM 8th: tumor-node-metastasis stage of 8th edition for hepatocellular carcinoma; ICC-TNM 8th: tumor-node-metastasis stage of 8th edition for intrahepatic cholangiocarcinoma; OS: overall survival.
